# Behavior Rating Inventory of Executive Function in Preschool (BRIEF-P) and Attention-Deficit and Hyperactivity Disorders (ADHD): A Systematic Review and Meta-Analysis of Floor and Ceiling Effects

**DOI:** 10.3390/children11010058

**Published:** 2023-12-30

**Authors:** Esperanza Bausela-Herreras, Yurena Alonso-Esteban, Francisco Alcantud-Marín

**Affiliations:** 1 Department of Health Sciences, Public University of Navarra, 31008 Pamplona, Spain; esperanza.bausela@unavarra.es; 2 Faculty of Social and Human Sciences, University of Zaragoza, 44003 Teruel, Spain; yalonso@unizar.es; 3 Department of Developmental and Educational Psychology, University of Valencia, 46010 Valencia, Spain

**Keywords:** BRIEF-P, floor effect, ceiling effect, executive functions, ADHD, test, diagnostic validation

## Abstract

Background. ADHD is a neurodevelopmental disorder that is accompanied by executive challenges. Objectives. To obtain evidence of the usefulness of the BRIEF-P and to analyze the possible ceiling and floor effect of its scores in the assessment of executive function in preschoolers with signs compatible with a possible diagnosis of ADHD. Method. A search was performed in Science Direct, NCBI (PubMed), and ProQuest Education Journals during the period 2012–2022. We included studies that evaluated samples of individuals with symptomatology compatible with ADHD, with an age range between 2 and 6 years, published in English or Spanish. Of a total of 2538 articles, only seven met the inclusion criteria. The risk of bias was assessed using the QUADAS-2 questionnaire. The main variables were age and executive functioning. Conclusions. Executive deficits in early-age individuals with symptoms compatible with ADHD are more extensive than just deficits in working memory. A floor effect has been found in tests associated with hot executive functions and a ceiling effect in cold executive functions. This makes it necessary to use different tests to assess executive performance in preschoolers with ADHD-compatible symptomatology and to design intervention proposals accordingly. The BRIEF-P is an instrument that facilitates obtaining a sensitive and discriminative executive profile, although it should be used in combination with other neuropsychological performance tests.

## 1. Introduction

Attention Deficit Hyperactivity Disorder (ADHD) is one of the most common neurodevelopmental disorders, and its prevalence in children is estimated at 2–7% [1]. Although there are very old precedents for its description in the literature (see [2]), its current concept dates back to the publication of the DSM-IV-TR [3]. The most characteristic symptoms are inattention (daydreaming), forgetting or losing things, motor restlessness, excessive talking (without control or respecting the turn of the conversation), making careless mistakes or committing recklessness, problems in relationships with others, etc. In the context of ADHD, daydreaming is often associated with inattention. It is a symptom not to be confused with “cognitive disengagement syndrome” (CDS), which has been replaced by “sluggish cognitive tempo” (SCT). SCT involves a constellation of behaviors involving excessive daydreaming, confusion and mental fogginess, and slowness of behavior and thinking. SCT is a distinct syndrome [4], although much work and research remain to clarify various aspects, such as its nature.

The DSM 5 [5] establishes as a criterion the presence of a persistent behavioral pattern of inattention and/or hyperactivity/impulsivity that interferes with functioning or development. In DSM 5 TR [6], up to three diagnostic subcategories are established depending on the combined presence of inattention and hyperactivity. The diagnostic criteria for ADHD in the DSM-5-TR remained identical to those in the previous edition. Koutsoklenis and Honkasilta [7] make a critique of these criteria, concluding that they are ambiguous, redundant, and arbitrary, reinforcing normality and inadequate attention to context.

Because of the nature of ADHD (multicausal etiology and heterogeneous symptoms [8,9]), different psychometric tools useful in the diagnostic process are cited in the scientific literature, according to DSM-5 criteria (e.g., Conners 3rd Edition [10]; ADHD Rating Scale-5 for Children and Adolescents [11], among others).

Many of the symptoms of ADHD are linked to different impairments of executive function (EF): inhibition, self-regulation, working memory, and flexibility, among others [12,13,14]. However, given the heterogeneity of ADHD, its high co-morbidity with other disorders and difficulties [15,16,17], and the different conceptions of the EF, there is no consensus on the existence of a characteristic profile [18,19,20]. Thus, the assessment of EF is taken as complementary information aimed both at excluding another disorder and at planning interventions.

Most studies on EFs consider the Miyake et al. [21] model as a reference, emphasizing cold EFs: inhibitory control [22], cognitive flexibility (or set shifting) [23], and working memory [24], among others. There is also a more-or-less-general consensus that the development of EFs starts during the first years of life and accelerates with schooling [25,26]. Evidence suggests that it is during the school period (6 to 12) that the development of hot and cold EFs is most evident [27]. Hot EFs, which require the intervention of affective regulation processes, include inhibitory control, decision making, and emotional regulation. Among the cold EFs, they are linked to reasoning, MT, attention, cognitive flexibility, and planning [28].

Some studies reveal the possibility of a slower development of hot EFs compared to cold EFs [29,30]. Rastikerdar et al. [31] compare the development of cold and hot executive functions in people with and without ADHD, pointing out differences in their development throughout the life cycle. Thus, cold EFs reach adult levels by the age of twelve, while hot EFs progress more slowly at school age and improve throughout adolescence. Shakehnia et al. [32], after controlling for age, parental education, and number of children, report significant differences between group means (hyperactivity disorder versus ADHD) in EF assessed with BRIEF (Behavior Rating Inventory of Executive Function): hot executive function (Behavioral Regulation Index), cold executive function (Metacognitive Index), total executive function, and other subscales. These researchers consider that hot executive functions and cold executive functions are related and interact, but that in people with ADHD, they are dissociated. Several studies have found a delay in the development of EF in people with ADHD [33,34]. This delay is not homogeneous or equal in all dimensions. Delays have been found in: (i) inhibitory control [33], (ii) working memory [35]; (iii) cognitive flexibility [36]; and (iv) delayed gratification and risky decision-making [37]. In conclusion [35], people who develop ADHD present a wide range of cognitive deficits, but they are only manifested in some EFs (fundamentally, cold EFs; see model by Zelazo and Carlson [28]). Several prospective studies have shown in individuals with ADHD the association of executive function deficits with poorer academic performance [38], the occurrence of substance use disorders [39], and other psychiatric comorbidities [40].

In general terms, it is accepted that the main impairment in people who develop ADHD is a deficit in inhibitory control (cold executive function) [33,41,42,43,44], to such an extent that Barkley [45] proposes the self-regulation model, understanding that the basic problem of people with ADHD is a deficit in behavioral inhibition which, in turn, has repercussions on other parallel cognitive and psychological processes: non-verbal working memory, verbal working memory, planning and reconstruction, and the self-regulation of emotions, motivation and arousal. Barkley’s model has been studied and compared with others that also consider self-regulation as the core deficit of ADHD [46]. However, symptom variability poses irresolvable problems for a model based predominantly on a single factor of EF. Evidence suggests that deficits in working memory [27,47,48,49,50] and deficits in cognitive flexibility (cold executive function) are also present in individuals who develop ADHD. A lack of adaptive capacity (cognitive flexibility) allows individuals to adapt their cognitive processing strategies to cope with unforeseen events [51], and its deficit correlates with mental health problems [52]. From a more organismic point of view, the results of studies based on brain imaging using functional magnetic resonance imaging (fMRI) show that the anterior cingulate, as well as the superior and inferior frontal gyri, which are involved in cognitive flexibility, are less active in people with ADHD [53], confirming the previous point.

Although the scientific literature shows a high level of interest in EF, its assessment remains complex and there is no consensus on the type of measure to use. The complexity of measuring EF lies in the different conceptual models [54] and in the need to perform a task where EF performance is evidenced. Some of the measures used, such as the Stroop Test, the Tower of Hanoi, or the Wisconsin Card Sorting Test, have low ecological validity and are not representative of the individual’s functioning in real-life contexts [55,56,57,58,59,60,61]. Based on these measures and sometimes benefiting from technological resources, digitized test batteries such as CANTAB (Cambridge Neuropsychological Test Automated Battery, [62]) have been developed. These include the NIH EXAMINER (Executive Abilities: Measures and Instruments for Neurobehavioral Evaluation and Research [63]), the CAS (Cognitive Assessment System [64] and CAS2 [65]), the D-KEFS (Delis–Kaplan Executive Function System [66]), and the CEFI (Comprehensive Executive Function Inventory [67]), among others.

However, these systems are complex to implement at school age due, among other reasons, to the high number of evaluation requests. An alternative is questionnaires based on rating scales such as the BRIEF (Behavior Rating Inventory of Executive Function; [68]). BRIEF is one of the most widely used scales in ADHD diagnosis and intervention. It is a family of questionnaires that attempt to capture the behavioral manifestations of executive dysfunction across the lifespan (2 to 90 years). The original BRIEF [68] has a range of application from 5 to 18 years of age, with separate forms for parents and teachers. There are self-report versions for adolescents (11–18 years) (BRIEF-SR [69]) and for adults (BRIEF-A [70]) from 18 to 90 years with separate self-report or third-person forms. As mentioned above, ADHD presents the first symptoms at early ages and has a need for valid and reliable tools at early stages in order to plan early intervention [71], so it is worth highlighting the preschool version of the BRIEF.

BRIEF-P (Behavior Rating Inventory of Executive Function-Preschool Version [72]) is an assessment tool adapted to Preschool Age (designed for children aged 2 to 5 years). The questions and scenarios are tailored to the understanding and experience of young children. BRIEF-P uses reports of observed behavior in everyday situations, providing a more complete and contextualized picture of the child’s executive skills in real-life environments. BRIEF-P results can be useful for planning specific interventions that address the identified areas of difficulty.

The assessment and diagnosis of ADHD are clinical and should be conducted by trained mental health professionals, and BRIEF-P is a tool that can complement the comprehensive assessment of the child. Additionally, the information gathered through BRIEF-P should be interpreted in the context of a broader evaluation that includes clinical observation, history collection, and other assessment methods.

Although there is evidence for the psychometric goodness of the BRIEF [73] and in particular of the BRIEF-P [74], at the clinical level the differentiation between typically developing children and ADHD is made dependent on the combination of the cut-off points of the test [75]. Examining the floor and ceiling effects of the BRIEF-P in clinical use is crucial for several reasons. Firstly, understanding these effects allows for assessing the instrument’s sensitivity to detect both deficiencies and exceptional abilities in executive function within the target population. Additionally, it helps to identify potential limitations of the instrument in its ability to discriminate adequately between extreme levels of performance. This information is essential for an accurate interpretation of results and informed decision-making in the clinical setting. By addressing floor and ceiling effects, we can enhance the utility and validity of the BRIEF-P as an assessment tool for preschool-aged children with neurodevelopmental disorders.

The aim of this paper is to determine, through an analysis of the scientific literature, the floor and ceiling scores of the EF components measured by the BRIEF-P in a population with symptoms compatible with ADHD.

## 2. Method

This systematic search and meta-analysis was registered in the Prospero platform under the registration number CRD42023465855. Articles in Spanish and English published in the databases Science Direct, NCBI (PubMed), and ProQuest Education Journals were reviewed. The articles had to be published between 2013 and 2023. In the databases, the search was restricted to “all article” and “journal article” in psychology, health, and education journals. The keywords were Attention Deficit Hyperactivity Disorder or ADHD or ADHD; Behavior Rating Instrument of Executive Function—School or BRIEF-P.

The research question was formulated following PICOS (Population, Intervention, Comparison, Outcome, Study Design) in an attempt to delve into the floor effect and ceiling effect of the BRIEF-P as an instrument to be considered in the early identification of symptoms compatible with ADHD:

Regarding the floor effect (minimum score), are there statistically significant differences in the executive profile of preschool children with ADHD versus typically developing children?

In terms of the ceiling effect (maximum score), are there significant differences in the executive profile of preschool children with ADHD versus typically developing children?

### Search Strategy

In order for studies to be included in the review, they had to meet the inclusion/exclusion criteria set out in Table 1. The methodological structure of the search and screening methods followed the guidelines of the PRISMA guide (Preferred Reporting Items for Systematic Reviews and Meta-Analyses [76,77]). The expressions of the searches were different for each database according to the database conditions, as shown below:

(i) Science Direct ◊
  - (Attention deficit hyperactivity disorder or ADHD) and (Behavior Rating Instrument of Executive Function-Preschool).
  - (Attention deficit hyperactivity disorder or ADHD) and (BRIEF-P).
(ii) NCBI ◊ PubMed Central ◊ 247 results
  - (Attention deficit hyperactivity disorder + or + ADHD) and (BRIEF-P).
(iii) ProQuest Education Journals
  - ((Attention deficit hyperactivity disorder or ADHD) AND (BRIEF-P)).
    AND stype.exact(“Scholarly Journals”) Education Database.
  - (Attention deficit hyperactivity disorder or ADHD) and (Behavior Rating Instrument of Executive Function-Preschool) Education Database.

**Figure 1:**
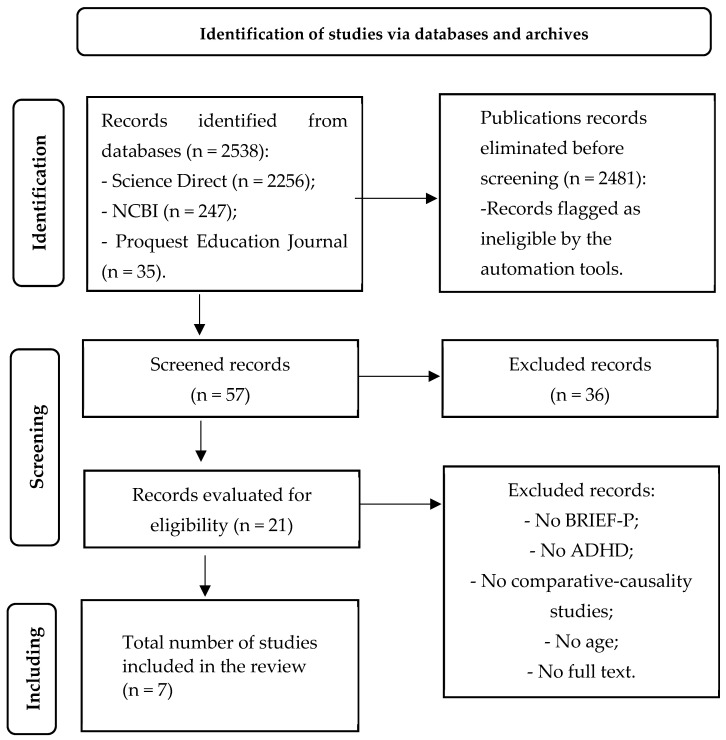
PRISMA flow diagram (based on the work of [76]).

After analyzing the 2538 papers, only 7 papers were selected that met the inclusion-exclusion criteria (see Table 1). The resulting flow can be visualized in Figure 1.

## 3. Results

A meta-analysis was conducted, continuing with the raw data available in each of the publications. A random-effects model was chosen for a meta-analysis. The random-effects model operates on the premise that the true effect may vary from one study to another due to differences (heterogeneity) among studies [77].

Table S1 (see Supplementary Materials) summarizes the characteristics of the seven studies included in the review with participants under 6 years of age with ADHD-compatible symptoms compared to typically developing participants, with executive functioning being assessed using BRIEF-P and with data on mean scores obtained by the ADHD group versus the typically developing control group. Table 2 shows the data relating to the number of participants in each study according to diagnosis.

### 3.1. Risk of Bias

We followed the PRISMA 2020 checklist for structured abstracts: Title, Background, Methods, Results, Discussion, and Other. To assess the methodological quality of the studies, we used the criteria outlined in Quality in Prognosis Studies (QUIPS), which consider six specific domains: sample selection, representativeness of the sample, measurement of variables, measurement bias, follow-up bias, and statistical analysis.

### 3.2. Floor Effect and Ceiling Effect

In the case of ADHD, variability in executive functioning can be significant among individuals. The main reasons to take into account the floor or ceiling effect in this sample are (i) limitations in measurement sensitivity: the floor effect occurs when most participants score very low, indicating that the measure used may not be sensitive enough to capture variations in performance; (ii) limitations in variance: the ceiling effect occurs when most participants score very high, suggesting that the measure may not be able to distinguish adequately between higher levels of ability or performance; (iii) impact on test validity: if a test is not able to accurately measure both lower and higher levels of the variable it is assessing, it can impact the overall validity of the test; and (iv) reflection of sample diversity: the presence of floor or ceiling effects may indicate the need for a more appropriate measurement tool, especially if the sample includes individuals with a wide range of abilities or levels of functioning.

Considering these effects is essential to ensure that the measure used is valid and sensitive to variations in performance within the studied sample.

A comparison was made between the group with symptoms compatible with ADHD and the typically developing group, considering the lower and upper scores, to find out the floor effect and the ceiling effect. Effect size estimates were calculated, taking Cohen’s d, the random effects model, as the effect size measure.

The results are presented in a forest plot or effects graph in which the results obtained in each study, as well as the overall effect and its 95% confidence interval, are presented in vertical order according to a certain criterion (the study number). The studies with lower precision are those that have greater visual impact (longer horizontal lines due to the greater confidence interval), and the mean value of each study is usually represented by a symbol (square or diamond) whose area is proportional to the weight with which the study intervenes in the overall calculation (inversely proportional to the variance), highlighting the most precise studies.

#### 3.2.1. Floor Effect

The studies with the lowest mean score obtained after the application of the BRIEF-P were selected. As a clinical instrument, higher scores indicate greater executive dysfunction.

The 10th percentile was chosen, placing the mean score between the following values: ADHD group (15–26) and typically developing group (13–20) Five studies obtained mean scores on the Flexibility clinical scale and on the Emotional Control index. The studies included in the meta-analysis were the mean score in the ADHD-compatible symptoms group [14,26] and the mean score of the typically developing group [12,32]. Significant differences were observed between the ADHD group and the typically developing control group (d = 0.615; Z = 4.335; *p* ≤ 0.001; 95% CI 0.337–0.893) (Table 3). With d = 0.615, we are in the moderate range. This suggests that the observed difference has practical significance but is not substantial enough to be considered a large effect. It may indicate that, although there is a significant difference, it might not be substantial enough to have a dramatic or practical impact.

**Figure 2:**
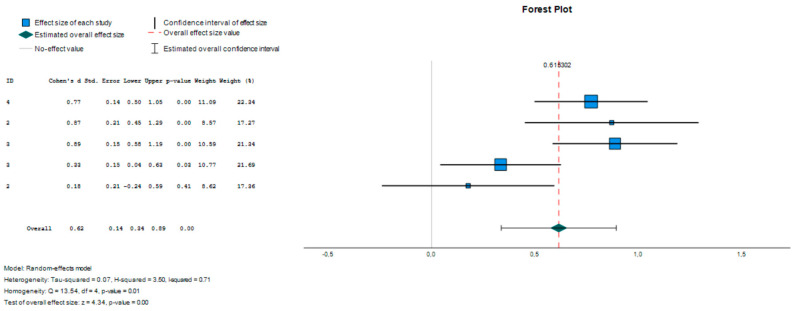
Forest plot (soil effect).

The studies included in the meta-analysis are represented in a forest plot (Figure 2). The study with the highest impact is study 4 and the study with the lowest impact is study 2.

#### 3.2.2. Ceiling Effect

The studies with the lowest scores were selected. Thus, studies with scores above the 90th percentile were selected, placing the mean score between the following values: ADHD group (100–108) and typically developing group (54–84). Four studies obtained mean scores on the Flexibility clinical scale and on the Emotional Control index. The studies were included in the meta-analysis with the mean score of the ADHD group (84,103) and the score of the typically developing group (67,89).

Significant differences were observed between the group with symptoms compatible with ADHD and the typically developing control group (d = 2.962; Z = 3.249; *p* ≤ 0.001; CI 95% 1.175–4.749) (Table 4). Such a large effect size suggests a noteworthy and practically significant difference between the compared groups.

**Figure 3:**
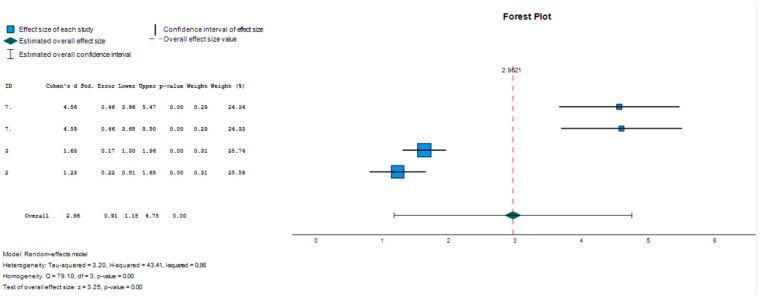
Forestry graph (soil effect).

The study with the highest impact is study 3 and the study with the lowest impact is study 7. The studies included in the meta-analysis are represented in a forest plot (Figure 3).

## 4. Discussion and Conclusions

The executive deficits presented by individuals in the early stages of the life cycle with symptoms compatible with ADHD are broader than deficits in working memory. There is a need for comprehensive assessment protocols for all dimensions that make up EF in line with the model of Miyake et al. [21] and adapted for children by Wiebe et al. [83] and the model developed by Zelazo and Carlson [28]. The results of the assessment will be the key to developing a personalized intervention [84]. With this review, we aimed to obtain evidence of the usefulness of the BRIEF-P by analyzing its floor and ceiling effects in the assessment of executive function in preschoolers with signs compatible with a possible diagnosis of ADHD.

The floor effect was associated with the Flexibility clinical scale and the Emotional Control index, both hot executive functions according to the model proposed by Zelazo and Carlson [28]. The study with the highest impact was the study by Skogan et al. [80] and the one with the lowest impact was the study by Ezpeleta and Granero [79]. Like other researchers [85], they suggested that emotional dysregulation contributes to ADHD symptomatology and is prevalent in ADHD across the lifespan.

The ceiling effect was associated with two of the BRIEF-P indices: Global Executive Functioning and the Emergent Metacognition index, configured by the clinical scales Working Memory and Planning/Organization. These indices are associated with the cold dimensions of executive functioning proposed in the model of Zelazo and Carlson [28]. The study with the highest impact is the study by Zhang et al. [22], and the one with the lowest impact is the study by Çak et al. [71]. Preschoolers with early symptoms consistent with ADHD presented significant difficulties in BRIEF-P. These results are in line with previous studies in which the core domains of EF (response inhibition [86,87], working memory [88,89], and flexibility [36,53]) were closely associated with ADHD symptoms.

In the mentioned results, three studies have been identified as reporting the floor effect in the BRIEF-P [22,79,80]. This implies that in these studies, participant scores were predominantly concentrated at lower levels of ability, suggesting that the instrument may not be sufficiently sensitive to detect differences in lower-level executive function skills in preschool children.

On the other hand, two of these studies identified reported the ceiling effect in the BRIEF-P [22,79]. This indicates that in these studies, participant scores were mainly concentrated at higher levels of ability, suggesting that the instrument may not be sensitive enough to differentiate between higher-level executive function skills in these preschool children.

Both floor and ceiling effects can be indicative of limitations in the test’s sensitivity to accurately measure the full range of abilities in the evaluated population. This may impact the test’s ability to detect real changes in performance over time or between different groups.

In summary, these results highlight potential limitations in the sensitivity of the BRIEF-P to accurately measure and differentiate between different levels of executive function skills in preschool children, both at lower and higher levels. This may necessitate adjustments to the instrument to enhance its assessment capability across the full spectrum of skills.

The findings obtained suggest potential limitations in the sensitivity of the BRIEF-P, prompting the consideration of several improvement actions for its strengthening: (i) Conduct a thorough review of the BRIEF-P design to identify possible areas for improvement. This may involve evaluating and adjusting items that could be contributing to the floor and ceiling effects. (ii) Consider the inclusion of new items that more effectively address the diversity of executive function skills in preschool children. This could help broaden the measurement range and enhance the test’s sensitivity. (iii) Carry out additional validation studies to confirm the effectiveness of the proposed modifications. This may include pilot testing with representative samples of the target population to ensure that the revisions have the desired impact. (iv) Consider adjustments to the scoring scale or interpretation of results to mitigate floor and ceiling effects. This could involve revising skill classification criteria to more accurately reflect the performance of preschool children. (v) Establish a continuous monitoring system to assess the effectiveness of the implemented modifications. This will allow for additional adjustments as needed and ensure the ongoing improvement of the BRIEF-P.

By implementing these recommendations, the goal is to strengthen the BRIEF-P’s ability to accurately measure executive functions in preschool children and overcome the identified limitations.

Studies suggest that children with ADHD exhibit statistically significant and large working memory deficits relative to their typically developing peers; however, executive deficits, which are broader, affect several executive dimensions including, for example, emotional dysregulation, and need to be further explored. This requires a comprehensive assessment and subsequent intervention, accordingly.

The results obtained point to the need to work on cold executive functions in preschoolers with symptoms compatible with ADHD. These include working memory, attentional control, problem solving, cognitive flexibility, phonological fluency, semantic fluency, error detection, and the inhibition of automatic responses [90].

The review has some limitations, as it is based on studies with small sample sizes. The average age of the participants (before ADHD diagnosis) precludes considering the influence of factors related to developmental stage and maturity in the study of people with ADHD.

The study of EF in preschoolers involves greater difficulties. The structure of EF in preschoolers requires further study. Some studies suggest a single general factor of executive functioning, with these functions being more differentiated with age, and others propose two (inhibition, working memory), three (inhibitory self-control, flexibility, emergent metacognition) or four (hyperactive behaviors, attention problems, disinhibition behaviors, emotional regulation behaviors) factors.

Subtypes of ADHD also need to be considered in future studies. Follow-up with children assessed for the first time at preschool age, in additional diagnostic groups and in larger samples, together with multiple performance-based EF tests, would be necessary. For future studies, we plan to analyze the concordance between standardized and performance measures in the identification of executive difficulties in preschoolers with early symptoms compatible with ADHD.

Finally, temporal variability may be a limitation of the study. The studies were focused on the last decade. Study results may vary over time due to changes in clinical practice, technological advances, or changes in the population studied. A meta-analysis may not fully capture these temporal variabilities.

These results provide us with information on the executive dimensions to be strengthened in preschoolers with early symptoms compatible with ADHD and the tests to be included to complement the information obtained after the application of BRIEF-P. This review supports the clinical utility of the BRIEF-P as a measure of EF in preschoolers with ADHD-compatible symptoms in combination with performance-based neuropsychological tests, integrating two models: (i) the three domains proposed by Miyake et al. [21] and (ii) the hot versus cold executive functions model developed by Zelazo and Carlson [28].

## Figures and Tables

**Table 1:** Inclusion/exclusion criteria.

Inclusion Criteria	Exclusion Criteria
(a) Participants: persons with symptoms compatible with ADHD obtained from the application of standardized tests;	(a) Participants: not diagnosed with ADHD;
(b) Cognitive age that allows the application of BRIEF-P;	(b) Cognitive age: does not allow application of BRIEF-P;
(c) Cognitive competence (obtained through the application of standardized tests);	(c) Cognitive competence: not available;
(d) Executive dimensions: single construct or basic dimensions (flexibility, inhibition and working memory);	(d) Assessment instruments: non-standardized;
(e) Assessment instruments: standardized for assessing executive functions: hetero- and/or self-report;	(e) Type of studies: case study and review;
(f) Type of studies: empirical;	(f) Type of design: non-comparative-causal;
(g) Type of design: non-experimental, comparative-causal (group with ADHD-compatible symptoms versus group with typical development);	(g) Type of design: non-comparative-causal;
(h) Language: English and Spanish;	(h) Language: other;
(i) Other characteristics: full text.	(i) Other characteristics: abstract, full text not available.

**Table 2:** Number of participants according to diagnosis in each of the seven studies selected for the meta-analysisi (references in Table S1 Supplementary Material).

Study	Author/Year/Country	ADHD	CONTROL	ODD	ODD + ADHD
1	(Lacerda et al. 2020) Brazil [78];	24	55		
2	(Ezpeleta and Granero, 2015) Sapin [79];	23	538	51	10
3	(Zhang et al. 2018) China [22];	163	63		
4	(Skogan et al. 2015) Norway [80];	1134			
5	(Schneider, Ryan & Mahone, 2020) USA [81];	49	35		
6	(Perrin, Heller & Loe, 2019) USA [82];	45	48		
7	(Çak, Çengel, Gökler, Öktem & Taşkıran, 2017) Turkey [71].	21	52		
SUBTOTAL		1459	791	51	10
TOTAL		2311			

**Table 3:** Effect size estimates for the studies (floor effect).

	Effect Size	Standard Error	Z	Sig. (Bilateral)	Confidence Interval 95%		Prediction Interval 95% ^a^	
					Lower	Upper	Lower	Upper
Global	0.615	0.1419	4.335	<0.001	0.337	0.893	−0.343	1.574

^a^. Based on t-distribution.

**Table 4:** Effect size estimates for the studies (ceiling effect).

	Effect Size	Standard Error	Z	Sig. (Bilateral)	Confidence Interval 95%		Prediction Interval 95% ^a^	
					Lower	Upper	Lower	Upper
Global	2.962	0.9117	3.249	0.001	1.175	4.749	−5.679	11.603

^a^. Based on t-distribution.

## Data Availability

Data are contained within the article.

## Supplementary Materials

The following supporting information can be downloaded at: https://www.mdpi.com/article/10.3390/children11010058/s1, Table S1: Characteristics of the studies included in the review and meta-analysis with their respective bibliographic references.
