# Supplementary material for: Behavior Rating Inventory of Executive Function in Preschool (BRIEF-P) and Attention-Deficit and Hyperactivity Disorders (ADHD): A Systematic Review and Meta-Analysis of Floor and Ceiling Effects
Source: Children (Basel). 2023 Dec 30;11(1):58. doi: 10.3390/children11010058 (PMC10814211; doi:10.3390/children11010058)
Supplement: Supplementary file 1 [file children-11-00058-s001.zip › children-2789620-supplementary.pdf]

Table S1. Characteristics of the studies included in the review.

Number	Author/ Year/ Country	Design	Sample/n	Age (Mean,SE)	IQ* (Inteligence Quotient) (Mean,SE)	Diagnostic instrument_ADHD	Assessment instrument for EF	Executive profile	Results
1	(Lacerda, y otros, 2020) Brazil	Causal Comparison: ADHD vs. Control	ADHD=24 Control=55	ADHD= 5.58 (0.92) (years) N-ADHD= 5.46 (0.72) (years)	ADHD=69.88(15.87) N-ADHD= 76.64(17.20)	The Schedule for Affective Disorders and Schizophrenia for School Aged Children-Present and Lifetime Version (K-SADS-PL)	>BRIEF-P >Conners' Kiddle Continious Perfomance Test (K-CPT 2)	Inhibition	Neuropsychological tasks and parent reports of executive functions (EF) may not be sensitive enough to differentiate preterm and/or underweight preschoolers with and without ADHD.
2	(Ezpeleta & Granero, 2015) [79] Sapin	Causal Comparison:Control vs. ODD vs. ADHD vs. ODD+ADHD vs. Control	Control=538 ODD=51 ADHD=23 ODD+ADHD=10	Control=3.76(0.33) (years) ODD=3.87(0.30) (years) ADHD=3.74(0.33) (years) ODD+ADHD=3.69(0.31) (years)	Control=99.8(15.4) ODD=98.0(16.4) ADHD=91.9(21.0) ODD+ADHD=88.5(16.6)	The Diagnostic Interview for Children and Adolescents for Parents of Preschool and Young Children (DICA-PPYC)	>BRIEF-P >The Kiddie-Continuous Performance Test (K-CPT)	Clinical Scales and Indices (Global)	Executive functioning deficits assessed with a performance-based with a performance-based measure or behavioral descriptions are specific to children with ADHD, compared to those with ODD.
3	(Zhang, y otros, 2018) [22] China	Causal Comparison: ADHD vs. Control	ADHD=163 Control=63	ADHD=59.1(7.2) (months) Control=59.7(5.3) (months)	ADHD=7(3) Control=8(4) *Matrices	Diagnostic Infant and Preschool Assessment (DIPA)	BRIEF-P	Clinical Scales and Indices (Global)	Combined assessment of performance-based neuropsychological testing and BRIEF-P in preschoolers with ADHD shows deficits in many areas.
4	(Skogan, y otros, 2015) [78] Norway	Causal Comparison: ADHD vs. ODD vs. Ansiedad vs. Control	ADHD=1134	Total= 41.8(1.3) (months)	Global= 101.8(9.2)	The Preschool Age Psychiatric Assessment Interview (PAPA)	BRIEF-P	Inhibition Working Memory	Early symptoms of ADHD were related to parent-reported difficulties primarily in inhibition and working memory. Deficits in these two domains of EF characterize early forms of ADHD. The clinical utility of the BRIEF-P as a measure of EF in preschoolers with ADHD symptoms is supported.
5	(Schneider, Ryan, & Mahone, 2020) [80] USA	Causal Comparison: ADHD vs. Control	ADHD=49 Control=35	ADHD= 5(0.6) Control= 4.9(0.5) (years)	ADHD=108.4(11.6) Control=109.7(13.2)	>Conners' Parent – and Teacher - Rating Scale Revises – Long Form (CPRS-R) (CTRS-R)	BRIEF-P	Inhibition Flexibility Emotional Control Working Memory Planning/Organization	There are differences between evaluators on BRIEF-P. Parents rated more symptoms than teachers.
6	(Perrin, Heller, & Loe, 2019) [81] USA	Causal Comparison: ADHD vs. Control	ADHD=45 Control=48	ADHD= 61(6.6) (months) Control=58(6.2) (months)	ADHD= 98.6(16.4) Control= 109.7(13.8)	>Child Behavior Checklist (CBCL)	BRIEF-P	Clinical Scales and Indices (Global)	Early identification of impairment in children with ADHD symptoms is recommended, along with specific intervention.

7	(Çak, Çengel, Gökler, Öktem, & Taşkıran, 2017) [71] Turkey	Causal Comparison:ADHD vs. Control	Control=52 ADHD=21	Control=56.9(9.1) (months) ADHD=58.1(8.3) (months)	Control=109.54(33.79 ADHD=98.13(19.45)	>The Kiddie-Schedule for Affective Disorders and Schizophrenia-Present and Lifetime versión (K-SADS-PL) >The Conners' Parent Rating Scales – Revised / Short Form (CPRS-R/S)	BRIEF-P	Clinical Scales and Indices (Global)	Combined methods are suggested for a comprehensive assessment of preschoolers with inattentive and hyperactive/impulsive behavior.

*IQ is Obtained with the application of the Wechsler intelligence scales.

References

Çak, H. T., Çengel, S. E., Gökler, B., Öktem, F., & Taşkıran, C. (2017). The Behavior Rating Inventory of Executive Function and Continuous Performance Test in Preschoolers with Attention Deficit Hyperactivity Disorder. *Psychiatry Investigation*, 14(3), 260-270. doi:https://doi.org/10.4306/pi.2017.14.3.260

Ezpeleta, L., & Granero, R. (2015). Executive functions in preschoolers with ADHD, ODD, and comorbid ADHD-ODD: Evidence from ecological and performance-based measures. *Journal of Neuropsychology*, 9(2):258-270. doi:https://doi.org/10.1111/jnp.12049

Lacerda, B., Martínez, S., Franz, A., Moreira-Maia, C., Silveira, R., Procianoy, R., . . . Wagner, F. (2020). Does ADHD worsen inhibitory control in preschool children born very premature and/or with very low birth weight? *Trends in Psychiatry and Psychotherapy*, 42(4):340-347. doi:https://doi.org/10.1590/2237-6089-2019-0075

Perrin, H., Heller, N., & Loe, I. (2019). School Readiness in Preschoolers With Symptoms of Attention-Deficit/Hyperactivity Disorder. *American Academy of Pediatrics*, 144(2):e20190038. doi:https://doi.org/10.1542/peds.2019-0038

Schneider, H., Ryan, M., & Mahone, E. (2020). Parent versus teacher ratings on the BRIEF-preschool version in children with and without ADHD. *Child Neuropsychology*, 26(1):113-128. doi:https://doi.org/10.1080/09297049.2019.1617262

Skogan, A. H., Zeiner, P., Egeland, J., Urnes, A.-G., Reichborn-Kjennerud, T., & Aase, H. (2015). Parent ratings of executive function in young preschool children with symptoms of attention-deficit/-hyperactivity disorder. *Behavioral and Brain Functions*, 11, Article 16. doi:https://psycnet.apa.org/doi/10.1186/s12993-015-0060-1

Zhang, H., Shuai, L., Zhang, J., Wang, Y., Lu, T., Tan, X., . . . Shen, L. (2018). Neuropsychological Profile Related with Executive Function of Chinese Preschoolers with Attention-Deficit/Hyperactivity Disorder: Neuropsychological Measures and Behavior Rating Scale of Executive Function-Preschool Version. *Chinese Medical Journal*, 131(6), 648-656. doi:https://doi.org/10.4103/0366-6999.226893.
